# The Myth of Man the Hunter: Women’s contribution to the hunt across ethnographic contexts

**DOI:** 10.1371/journal.pone.0287101

**Published:** 2023-06-28

**Authors:** Abigail Anderson, Sophia Chilczuk, Kaylie Nelson, Roxanne Ruther, Cara Wall-Scheffler

**Affiliations:** 1 Department of Biology, Seattle Pacific University, Seattle, Washington, United States of America; 2 Department of Anthropology, University of Washington, Seattle, Washington, United States of America; University of Michigan, UNITED STATES

## Abstract

The sexual division of labor among human foraging populations has typically been recognized as involving males as hunters and females as gatherers. Recent archeological research has questioned this paradigm with evidence that females hunted (and went to war) throughout the *Homo sapiens* lineage, though many of these authors assert the pattern of women hunting may only have occurred in the past. The current project gleans data from across the ethnographic literature to investigate the prevalence of women hunting in foraging societies in more recent times. Evidence from the past one hundred years supports archaeological finds from the Holocene that women from a broad range of cultures intentionally hunt for subsistence. These results aim to shift the male-hunter female-gatherer paradigm to account for the significant role females have in hunting, thus dramatically shifting stereotypes of labor, as well as mobility.

## Introduction

The notion of separate subsistence roles for females and males in forager societies has been a long-standing paradigm for a wide range of social science disciplines, as well as in the public sphere, and include influential texts such as Man the Hunter [1], and Woman the Gatherer [2]. This viewpoint further purports that females engage in the majority of child-rearing activities, which is aligned with the slow-moving pace of gathering. Such assumed sex-specific gender roles in subsistence activities are commonly construed with additional gendered traits such as human men being less emotional and more aggressive, while human women tend to demonstrate more nurturing behavior and a focused interest in children. While we have known these patterns are culturally defined and thus variable for over a century now (e.g. [3–5]), it is only recently that the data are building to move a more accurate paradigm of subsistence flexibility into discourse [6–8].

One of the most prominent discoveries recently includes a 9,000 year old burial located in the Andean highland area of Wilamaya Patjxa in Peru [9]. The burial included an adult female alongside a hunting toolkit consisting of stone projectiles as well as animal processing equipment [9]. Researchers typically presume that stone projectiles buried alongside males are hunting tools but are less persuaded when projectiles are associated with females; the specific assemblage clearly evidenced hunting in this case. In their own review of the literature, Haas et al. [9] examined burials in the Americas from the Late Pleistocene to the Early Holocene period, identifying eleven females from ten sites who were associated with big-game hunting tools. By using a probability analysis of all twenty-seven sites which had evidence of big-game hunting, Haas et al. determined that females made up a “nontrivial” amount of big-game hunters across the Americas [9]. In fact, their analysis suggested that females represented up to fifty percent of big game hunters from the Americas prehistorically.

In addition to tools generally associated with big-game hunting being conferred to males, tools associated with warfare are also consistently assumed to occupy burials of males [10]. In 2017, a well-known burial in Sweden revealed an individual alongside weapons and equipment associated with high-ranking Viking warriors [11]. The individual was assumed to be male considering the historical interpretation of the prevalence of male warriors, but genomics confirmed that the individual was a female. In addition, archaeologists discovered a 2,500 year old burial site that contained four females associated with weapons and warrior equipment [12]. The age of the females ranged from 12 or 13 years old to 40 to 50 years old and were believed to be a part of the nomadic group known as Scythians [12]. Scythian women were warriors in their culture as supported by the fact that one-third of the females in this society were buried with weapons [12]. The purpose of these anecdotes is two-fold. Firstly, researcher bias shapes science’s interpretation of data, and it behooves each generation of scientists to ensure that paradigms fit the existing data. Secondly, the number of anecdotes on females taking up weapons and tools interpreted as “violent” is extensive across time as well as space [13, 14], making such examples more of a pattern of female behavior than anecdotal [10].

The discovery and reanalysis of the human burials from a range of geographic and temporal situations has prompted further research into the organizational structure of many Holocene societies. The question of the division of labor being sex (biological denotation, often based on external cues like genitalia) or gender (social denotation, often based on biological cues, but shaped by the intersection of social norms and personal expression) specific among human populations remains insufficiently researched and undetermined [7, 15]. Here we aim to close some of the research gap between female and male subsistence roles by gleaning, with as much resolution as possible, information on subsistence strategies among forager groups around the world. Our hypothesis is that the majority (i.e., more than half) of hunter-gatherer communities do expect females to contribute to hunting strategies. Such findings would continue the challenge to long-held perceptions of sex-specific gender roles within foraging subsistence labor [6, 7, 9, 10, 16].

## Methods

**Fig 1:**
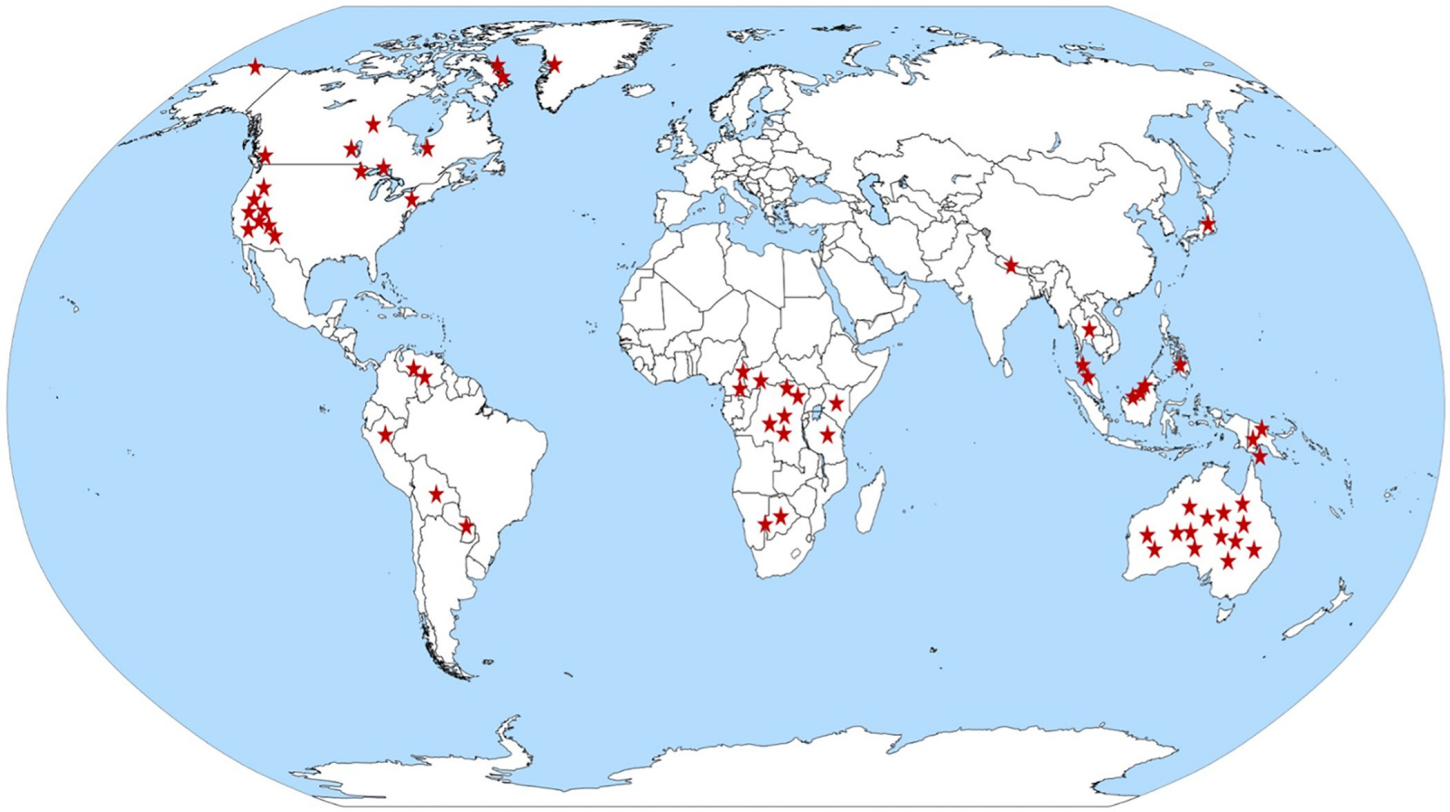
World map of the locations of 63 different foraging societies analyzed. The map is in the public domain and can be attributed to Petr Dlouhy, https://commons.wikimedia.org/wiki/File:World_Map_Blank_-_with_blue_sea.svg.

The relationship between subsistence activity and gender was compiled by reading ethnographic reports of foraging societies. A list of potential foraging societies along with their location and type of subsistence activity was first constructed using D-PLACE, the Database of Places, Languages, Culture and Environment [17]. This database is based on the ethnographic atlas by Lewis Binford [18] and contains detailed information on over 1,400 human societies. In order to reasonably sample across geographic areas, 391 foraging societies from around the globe were chosen to investigate further. Of the 391 different societies the continent, location, ecosystem, and primary subsistence activity were obtained from D-PLACE and recorded. Each foraging society was then investigated by searching through the original references cited in D-PLACE [17], Binford [18], and by searching digitized databases and archives. Multiple reports featuring the same foraging societies were read to ensure accuracy and reliability. Of the 391 foraging societies, explicit data on hunting was obtained for 63 of the societies (Fig 1; Table 1).

**Table 1:** Cultural groups. Foraging societies grouped by continent with foraging society name and approximate location. An asterisk denotes evidence of women hunting.

Continent	Cultural Group	References
**Africa (12)**	*!Kung San (Botswana)	Lee 1979; Singh 2001 [22, 23]
	*Ju/’hoansi (Botswana/Namibia)	Brightman 1996; Lee 1979 [22, 24]
	*Bakola (Southern Cameroon)	Ngima Mawoung 2006 [25]
	*Baka/BaYaka (Cameroon)	Reyes-García et al. 2020 [26]
	*Aka (Democratic Republic of the Congo)	Kitanishi 1995; Noss & Hewlett 2001 [27, 28]
	*Bambote (Democratic Republic of the Congo)	Terashima 1980 [29]
	*Mbuti (Democratic Republic of the Congo)	Brightman 1996; Ichikawa 1987 [24]
	*Efe Pygmies (Ituri Forest)	Brightman 1996; Peacock 1991 [24]
	*Sua/Tswa (Ituri Forest)	Bailey & Aunger 1989 [30]
	Kikuyu (Kenya)	Fedders & Salvadori 1988; Nyamanga n.d. [31, 32]
	*Hadza (Tanzania)	Marlowe 2010 [33]
	*Bofi (Southwest Central African Republic)	Lupa & Schmitt 2002 [34]
**Asia (5)**	*Ainu (Japan)	Goodman et al. 1985; Brightman 1996 [21, 24]
	*Batek De’ (Malay Peninsula)	Endicott 1984 [35]
	Jahai (Malay Peninsula)	Van der Sluys 1996 [36]
	Tamang (Rural Nepal)	Panter-Brick 1989 [37]
	Maniq (Thailand)	Lukas 2004 [38]
**Australia (15)**	*Adnjamatana (Australia)	Mountford & Harvey 1941 [39]
	*Alyawara (Australia)	Devitt 1988 [40]
	*Australian Mardudjara (Western Australia)	O’Dea 1991 [41]
	*Australian Martu (Western Australia)	Bird & Bird 2008 [42]
	Gunwinggu (Australia)	Gunwinggu 2015 [43]
	Kaiadilt (Australia)	Memmott et al. 2008 [44]
	Karajarri (Australia)	Willing 2014 [45]
	Kaurareg (Australia)	Boore 2004; Southon & Elders 2014 [46, 47]
	*Lardil (Australia)	Memmott et al. 2008; Lardil 2015 [44, 48]
	Larrakia (Australia)	Day 2012 [49]
	*Tasmania (Australia)	Tindale 1974 [50]
	*Tiwi (Australia)	Hart et al. 1988 [51]
	*Walbiri (Central Australia)	Tonkinson 1978 [52]
	*Worrorra (Australia)	Clendon 2014 [53]
**North America (19)**	*Basin-Plateau (North America)	Steward 1997 [54]
	*Belcher Island Eskimo (Eastern Arctic)	Guemple 1986; Brightman 1996 [24, 55]
	*Central Eskimo (Northeast America)	Boas 1888 [56]
	*Cree (James Bay in Ontario/Quebec)	Flannery 1995 [57]
	*Fish Lake Valley North Paiute (North America)	Steward 1997 [54]
	*Gosiute (Southwestern USA)	Steward 1997 [54]
	*Inuit (Eastern Arctic)	Guemple 1986 [55]
	*Iñupiaq (Barrow, Canada)	Bodenhorn 1990 [58]
	*Iroquois (Northeastern USA)	Brown 1970 [59]
	*Kalaallit (Greenland)	Issenman 1997 [60]
	*Maidu (Southwestern USA)	Faye 1923 [61]
	*Mescalero Apache (Southwestern USA)	Flannery 1932; Brightman 1996 [24, 62]
	*Missinippi Cree (Manitoba)	Brightman 1993; Brightman 1996 [24, 63]
	*Mono Lake Northern Paiute (Southwestern USA)	Lubinski 1999 [64]
	*Nootka (Pacific Northwest Coast in Canada)	Morris 1995 [65]
	*Northern Ojibwa (Lake Superior)	Landes 1938 Brightman 1996 [24, 66]
	*Rainy River Ojibwe (Northeastern USA)	Buffalohead 1983 [67]
	Tolowa (Southwestern USA)	Collins 2014 [68]
	Tongva (Southwestern USA)	Williams 2003 [69]
**Oceania (6)**	*Agta/Cagayan (Malesia)	Goodman et al. 1985 [21]
	*Ayta/Pinatubo (Malesia)	Goodman et al. 1985 [21]
	*Batak (Philippines)	Goodall 1971 [20]
	*Ganij (New Guinea)	Hewlett 1996 [70]
	Punan (Malesia)	Ryes-García & Pyhala 2017 [71]
	*Wopkaimin (New Guinea)	Hyndman 1984 [72]
**South America (6)**	*Hiwi (Southwest Venezuelan Llanos)	Hurtado & Hill 1990; Gurven & Hill 2009 [73, 74]
	*Matses (Peruvian Amazon)	Romanoff 1983 [75]
	*Northern Ache (Eastern Paraguay)	Hurtado et al. 1985 [76]
	Savanna Pumé (Southwest Venezuela)	Hilton & Greaves 2008 [77]
	*Tsimane (Bolivia)	Medinaceli & Quinlan 2018 [78]
	*Yamana (Southern Archipelago)	Martens 2016 [79]

Data used for this study included reports on what, when, and how hunting occurred in the cultural group. Ethnographic reports needed to include explicit information, in the form of tables or sentences that females went on hunting trips, and were involved in tracking, locating animals, and helping with the killing if applicable. Given that there is a difference between the phrase ‘women went hunting’ and ‘women accompanied the hunters’ it should be noted that we were looking for phrases along the lines of ‘women were hunting’ or ‘women killed animals,’ not references to the idea that women might be accompanying men “only” to carry the kills home, though obviously this does happen as well (e.g. [19]). Specific contributions such how much killing took place, and total calories from female-only kills were not written about frequently enough to warrant their assessment here.

If women were hunting, it was further investigated to see if the hunting was done purposely, whether women would go out with the intention to hunt, or whether women were hunting spontaneously (i.e. “opportunistically”); this might occur when women may have been doing a different task but if the opportunity arose, they would kill an animal. This was determined by explicit statements in the published literature or by a judgment based on the descriptions. Women’s involvement in hunting was determined by written documentation explicitly stating that women were hunting in that particular foraging society or were excluded and in some instances even forbidden to hunt. The most important subsistence activity was also compared to the relative frequency of women hunting. Additionally, the type of the game hunted was assembled into three categories of small, medium, and large. The type of game was defined by the relative size of the prey, hunting toolkit that was used, or if size was explicitly stated in the literature. For example, when looking at the Tiwi society of Australia, a study reported that Tiwi women regularly hunted small animals while the hunting of large game was a man’s activity, suggesting that women were involved in hunting small game only [20]. In instances where the type of game was not explicitly stated, it was determined from other clues in the report. For example, accounts of the Matses from the Amazon state that the women would strike their prey with large sticks and machetes, which would account for large game whereas other societies had documentation of small digging sticks or the killing of rodents, suggesting the prevalence of small game hunting [21]. The prevalence of women hunting with children and dogs was also recorded and analyzed based on statements in the literature.

Compiled data were analyzed to determine the frequency of females hunting, the type of hunting accomplished, as well as the relative size of game.

## Results

Data were compiled from literature on sixty-three different foraging societies across the globe. These included nineteen different foraging societies from North America, six from South America, twelve from Africa, fifteen from Australia, five from Asia and six from the Oceanic region (Fig 1 & Table 1). Of the 63 different foraging societies, 50 (79%) of the groups had documentation on women hunting. Of the 50 societies that had documentation on women hunting, 41 societies had data on whether women hunting was intentional or opportunistic. Of the latter, 36 (87%) of the foraging societies described women’s hunting as intentional, as opposed to the 5 (12%) societies that described hunting as opportunistic. In societies where hunting is considered the most important subsistence activity, women actively participated in hunting 100% of the time.

The type of game women hunted was variable based on the society. Of the 50 foraging societies that have documentation on women hunting, 45 (90%) societies had data on the size of game that women hunted. Of these, 21 (46%) hunt small game, 7 (15%) hunt medium game, 15 (33%) hunt large game and 2 (4%) of these societies hunt game of all sizes. In societies where women only hunted opportunistically, small game was hunted 100% of the time. In societies where women were hunting intentionally, all sizes of game were hunted, with large game pursued the most. Of the 36 foraging societies that had documentation of women purposefully hunting, 5 (13%) reported women hunting with dogs and 18 (50%) of the societies included data on women (purposefully) hunting with children. Women hunting with dogs and children also occurred in opportunistic situations as well.

## Discussion

Here we investigated whether noted trends of non-gendered hunting labor known from the archaeological record continued into more recent, ethnographic periods. The descriptive sample described here is sufficient to warrant the conclusion that women in foraging societies across the world participate in hunting during more recent time periods, a finding that makes sense given women’s general morphology and physiology [16, 80]. The prevalence of data on women hunting directly opposes the common belief that women exclusively gather while men exclusively hunt, and further, that the implicit sexual division of labor of ‘hunter/gatherer’ is misapplied. Given that this bimodal paradigm has influenced the interpretation of archeological evidence, which includes the reluctance to distinguish projectile tools found within female burials as intended for hunting (or fighting) [9, 10, 81], this paper joins others in urging the necessity to reevaluate archeological evidence, to reassess ethnographic evidence, to question the dichotomous use of ‘hunting and gathering,’ and to deconstruct the general “man the hunter” narrative [6, 7, 80].

Based on the data supporting the existence of female hunters, certain skills and practices within foraging societies allow women to be successful hunters. Of the 63 foraging societies with clear descriptions of hunting strategies, 79% of them demonstrated female hunting. The widespread presence of female hunting suggests that females play an instrumental role in hunting, further adding to the data that women contribute disproportionately to the total caloric intake of many foraging groups [15, 28, 82, 83]. Additionally, over 70% of hunting done by females is interpreted as intentional, meaning that females play an active and important role in hunting—and the teaching of hunting—even if they use different tools and employ different acquisition strategies. For example, among the Aka, women’s participation in net-hunting was required, whereas men’s participation was not [28].

These data suggest that females not only prepare to hunt and actively pursue game, but also that they are skilled in the practice. This is supported by both the existence of a specialized toolkit, as well as distinct strategies compared to their male counterparts, potentially relating to different training regimes, as well as different cultural norms surrounding the hunting, processing, and eating of meat (e.g., [24]). For example, the tools used by Agta women from the Philippines are remarkably different compared to Agta men [21, 84]. Whereas Agta men heavily rely on a consistent strategy of bow and arrows [84], women are much more likely to have personal preferences and show variation. Some women prefer hunting only with knives, a few women use bow and arrows, and others use a combination of the two [84]. Among the Aka, women are also flexible—carrying nets, but also spears, machetes, and cross bows. Even when nets are primarily used in hunting, sometimes women will wield the nets and sometimes men will wield the nets [28].

In addition to weapon choices, women further employ a greater flexibility of hunting strategies compared to men. For example, women hunt with a variety of partners, including their husbands, other women, children, dogs, as well as hunting alone [7, 21]. In contrast, men primarily hunt alone, with a single partner (their wife), or with a dog [18, 22]. Among the Agta, women might hunt in teams, and largely hunt during the day, though they might also hunt unaccompanied [84]. Agta men predominately hunt alone or with one other person if they are hunting at night in the forest [84]. Further, dogs are important to Agta women hunters, while the men typically only are accompanied by dogs when also hunting alongside women; the number of available dogs is a crucial factor in determining the frequency of Agta women hunters, in which a minimum of three mature dogs are typical for success [84]. Among the Aka, the size of the hunting net and the range of travel can depend on what else the women are carrying, whether a child is present, and whether they also have a basket [28].

As might be expected based on both tool and technique specializations, females maintain specializations for certain animals. American Cree women hunt pelt-animals alone and in groups [24]. Additionally, Mbuti women from the Congo hunt using nets [7], and Aka women also hunt using nets, more than men hunt using nets. The difference between these two populations is that among the Mbuti, women usually are flushing out the game, whereas among the Aka, women are usually capturing the game [28]. The Aché and Ju/’hoan women participate in hunting by tracking [7]. The Peruvian Matses and Mossapoula Aka women actively hunt with their husbands in order to increase overall hunting yields [7, 75]. Given that social norms determine how tools are made, and by whom [85], these specialized skills warrant much more attention by the literature. This would allow information on exactly who and how the tools are made, as well as to whom and how skills are being disseminated, can be used to uncover the means by which tasks are taken on by all the members of a group [86].

Suggestions that children are put in danger by accompanying hunts [74] can be mediated with current literature on the numerous ways in which infants and children are carried during expeditions by parents and alloparents. The importance of infants remaining with adults (versus being parked) is an important part of our lineage [87, 88], with children accompanying the wide range of expeditions consistently evidenced in the archaeological [89], as well as the ethnographic record [90]. Data explicitly mentioning that infants are carried while hunting exist for the Aka [91] and the Awa [92], as well as for foraging bouts that might result in opportunistic hunting (e.g., among the Batek [93] and Nukak [94]). Among both the Hadza and the Aka, children (potentially as young as age three) accompany adults on over 15% of hunting trips [95]. The idea that women are hindered by childcare and thus cannot hunt is an area where increasing data collection and thoughtful interpretation is lending a much richer lens to our understanding of human mobility strategies.

Women in foraging societies across the world historically participated and continue to participate in hunting regardless of child-bearing status. The collected data on women hunting directly opposes the traditional paradigm that women exclusively gather and men exclusively hunt and further elucidates the diversity and flexibility of human subsistence cultures [96]. Because the hunter/gatherer paradigm has prevented the recognition of contributions by women to hunting, a new framework would enable past and future discoveries to be evaluated in the context of female hunters. Furthermore, the term “forager,” as suggested by Brightman [24], should be used to acknowledge the non-sexual division of labor concerning hunting and gathering, in order to develop an inclusive framework for understanding human culture [9].

## Supporting information

S1 Table Data table. This is the data table used for this analysis.
(XLSX)
