# Correction: The Myth of Man the Hunter: Women’s contribution to the hunt across ethnographic contexts

**DOI:** 10.1371/journal.pone.0309543

**Published:** 2024-08-22

**Authors:** Abigail Anderson, Sophia Chilczuk, Kaylie Nelson, Roxanne Ruther, Cara Wall-Scheffler

After publication of this article [1], concerns were raised about the reliability and replicability of the results. The authors report that while some of the concerns raised reflect ongoing, open questions in the field, some aspects of the methodology could benefit from more detail.

This notice seeks to provide additional nuance and methodological information to further conversations about this topic.

The data on hunting and how these data were identified and extracted from D-PLACE did not initially include page numbers. The updated data table with page numbers included has now been provided as Supporting Information with this notice.

## Additional Methodological Information

### Definition of “forager”

The word ‘forager’ is often used by anthropologists to imply a member of a small-scale subsistence group who gains access to diverse or varying foods around a daily or seasonal basis. It can imply a highly mobile lifestyle, but also does not have to, as some foragers are referred to as ‘semi-sedentary foragers’ [2]. A broad use of the word forager is not atypical; small-scale “horticulturalists” or “agriculturalists” who also hunt and gather (as do the groups in this study) are regularly included under the umbrella of “forager.” The term foraging is considered to imply small-scale subsistence, and to include hunting (even when the group also uses horticulture) [3]. Whereas people might have personal preferences about the term that is used, the majority of anthropologists see the word forager as inclusive of small-scale societies that practice a wide range of seasonally varying activities that can include hunting, gathering, horticulture, and agriculture [4–6]. These are not rigid boxes for small human populations and we continue to be comfortable with the broad reaching meaning of forager which includes small-scale groups who participate in seasonal foraging and hunting, as well as seasonal production of some crops. Regardless, concerns that the diversity of groups included in Anderson et al.’s study might have skewed the results are unwarranted as the five groups identified as being of a broader definition of foraging have the exact same percentage as the group as a whole, namely 80% show evidence of women killing terrestrial vertebrates.

### Selection criteria for the included societies

The groups were chosen because books or papers about them relating to subsistence economy were identified in D-PLACE. D-PLACE was initially developed as a place to identify linguistic groups, and as such only a small fraction of all the groups had initial indications that subsistence information was known for the groups. Once we had the smaller dataset of groups with known subsistence strategies, we began the work of determining which groups had further information about specific food acquisition methods. This involved looking for books or papers that referenced these groups. We used the references listed in D-PLACE, Binford’s ethnographic atlas [7] and electronic search engines (university libraries, Web of Science, JSTOR, Google) to find references to papers, books or monographs that mentioned the named subsistence groups. If the group did not actually have a reference listed in D-PLACE, or if we could not find any mention to a group’s subsistence in an electronic database (including JSTOR which searches digital archives and not just key words) then that group was not included further in the study. Included groups, at this stage, were those where literature was found containing data on how the group used the land and food/water resources; these groups represented all habitable continents. Sometimes the groups would be referenced in works cited but the citations were to volumes not available electronically, so those books/papers/monographs were obtained from libraries. All material, whether available electronically or in hard copy, was read cover-to-cover. We did not use electronic word searches. If data on how the group got food was not available, they were excluded. The next step was identifying the gender of the hunters, which determined the final sample size reported in the article. If the gender of the hunters was not available, the group was excluded.

### Procedure for assessing the content and reliability of the included research reports

Primary sources or work by the initial ethnographer (as determined by the earliest citations) of a particular geographic area whose work was the main source of information on a particular group, as cited by others, were prioritized. In most cases all papers and articles about subsistence written by the initial or most cited ethnographer were read until identification of the gender of the person or people doing the hunting was identified. Ethnographies typically refer to the ‘hunters’ or the ‘foragers’ or some general phrase. Any time the gender of people killing animals was specified, the group was included in the sample. When the ethnographer would specifically refer to men as hunters, or would provide data of hunts being done by men, and also provided clear explanations of what women were doing that was not hunting, we considered this as evidence for groups in which hunting was only practiced by men. Sometimes these studies also provided further information about taboos against women hunting or at least taboos against using the tools that would have made hunting more straight forward. When there were tables that outlined what was hunted by whom, they were examined to determine what genders were represented in the Table. When there were no tables, data were typically presented in prose form that explained who provided what kind of food during which season. If all the meat across all seasons was listed as being provided by men, then that group was classified as a group that did not have women hunters. When women were included as providing meat through killing, then that was considered a group in which women hunted. A table of subsistence data and gender of provider was the stop point. If there was no table, then the explanation of who collected which food groups was the stop point. The supplementary table includes the page numbers for primary sources and initial/primary ethnographers.

### Method for determining hunting behavior when not explicitly stated

Descriptions of killing terrestrial or avian vertebrates was considering hunting behavior, even when the word used by ethnographers was not “hunt.” Words that might describe killing such creatures included kill, spear, shoot, trap, snare, drive, forage, track, stalk, or acquire. The words describing the prey was key in these circumstances, for example, “foraging for rabbits,” “driving caribou,” or “acquiring goanna lizards.”

### Method for comparing the most important subsistence activity to the relative frequency of women hunting

The main annual subsistence activity for a group of people reported by ethnographers was recorded. The groups that had a ‘main’ subsistence activity were compared to whether women were likely to be identified as hunters in that group. These data were used in the last sentence of the first paragraph of the results.

### The method for determining game size categories

The assessment of size was based on how the ethnographers identified the game, given that size of animals varies in different parts of the world (e.g. Bergmann and Allen rules). If the ethnographer didn’t identify it, but used the word ‘rodent’ or ‘rabbit’ then we designated it as small. If the ethnographer didn’t identify it, deer and pigs were designated large. Certain lizards and baby animals would be medium, or if the ethnographer identified the deer as medium (i.e. in SE Asia), then these would be medium.

### The method for determining game type when not explicitly stated

Ethnographers typically identify the size of the prey, or use the labels as above with rodents, rabbits, and small lizards (<10kg) being small, large lizards, turtles, and baby ungulates/equids being medium (20-30kg), and adult pigs and ungulates being large (>50kg).

The authors hope these additional methodological details clarify the published article and inspire future scholars.

## Supporting information

S1 Table Updated Data Table. This is the data table used for analysis in [1], updated to include page numbers.
(XLSX)
